# COVID-19 and Antimicrobial Resistance: Data from the Greek Electronic System for the Surveillance of Antimicrobial Resistance—WHONET-Greece (January 2018–March 2021)

**DOI:** 10.3390/life11100996

**Published:** 2021-09-22

**Authors:** Michalis Polemis, Georgia Mandilara, Olga Pappa, Athina Argyropoulou, Efstathia Perivolioti, Nikolaos Koudoumnakis, Spyros Pournaras, Alexandra Vasilakopoulou, Sophia Vourli, Helen Katsifa, Theodoros Karampatakis, Anastasia Papavasiliou, Efthymia Petinaki, Stylianos Xitsas, Lemonia Skoura, Efthymia Protonotariou, Paraskevi Mantzana, Konstantina Gartzonika, Efthalia Priavali, Amalia Kallinteri, Panagiota Giannopoulou, Nikoletta Charalampaki, Meletis Memezas, Zervaki Calina Oana, Marina Papadogianni, Maria Panopoulou, Athanasia Koutsidou, Alkiviadis Vatopoulos, Kyriaki Tryfinopoulou

**Affiliations:** 1 Central Public Health Laboratory, National Public Health Organization, 16672 Vari, Greece; o.pappa@eody.gov.gr (O.P.); k.tryfinopoulou@eody.gov.gr (K.T.); 2 School of Public Health, University of West Attica, 11521 Athens, Greece; gmandilara@uniwa.gr (G.M.); avatopoulos@uniwa.gr (A.V.); 3 “Evaggelismos” General Hospital, 10676 Athens, Greece; athina.argyropoulou@gmail.com (A.A.); perivolioti@yahoo.gr (E.P.); nikoskoudou@hotmail.com (N.K.); 4 “Attikon” University Hospital, 12462 Athens, Greece; spournaras@med.uoa.gr (S.P.); dralexandravasilakopoulou@gmail.com (A.V.); svourli@med.uoa.gr (S.V.); 5 General Hospital “George Papanikolaou”, 57010 Thessaloniki, Greece; elen02micro@yahoo.gr (H.K.); tkarampatakis@yahoo.com (T.K.); natpap83@gmail.com (A.P.); 6 University Hospital of Larissa, 41110 Larissa, Greece; petinaki@uth.gr (E.P.); stexit007@gmail.com (S.X.); 7 “Axepa” University Hospital, 54636 Thessaloniki, Greece; lemskour@auth.gr (L.S.); protonotariou@auth.gr (E.P.); vimantzana@gmail.com (P.M.); 8 University Hospital of Ioannina, 45500 Ioannina, Greece; kgartzon@uoi.gr (K.G.); epriaval@gmail.com (E.P.); akallinteri@yahoo.gr (A.K.); 9 “Thriasio” General Hospital of Elefsina, 19600 Athens, Greece; giogianop@gmail.com (P.G.); jijouxani@yahoo.gr (N.C.); mmemezas@yahoo.gr (M.M.); 10 “St. George” General Hospital, 73300 Crete (Chania), Greece; zervakiskal@gmail.com (Z.C.O.); marinapa2dogianni@gmail.com (M.P.); 11 University Hospital of Alexandroupolis, 68100 Alexandroupoli, Greece; mpanopou@med.duth.gr (M.P.); loimoxeis@pgna.gr (A.K.)

**Keywords:** antimicrobial resistance, COVID-19, routine laboratory data, surveillance system

## Abstract

Changes in hospitals’ daily practice due to COVID-19 pandemic may have an impact on antimicrobial resistance (AMR). We aimed to assess this possible impact as captured by the Greek Electronic System for the Surveillance of Antimicrobial Resistance (WHONET-Greece). Routine susceptibility data of 17,837 Gram-negative and Gram-positive bacterial isolates from blood and respiratory specimens of hospitalized patients in nine COVID-19 tertiary hospitals were used in order to identify potential differences in AMR trends in the last three years, divided into two periods, January 2018–March 2020 and April 2020–March 2021. Interrupted time-series analysis was used to evaluate differences in the trends of non-susceptibility before and after the changes due to COVID-19. We found significant differences in the slope of non-susceptibility trends of *Acinetobacter baumannii* blood and respiratory isolates to amikacin, tigecycline and colistin; of *Klebsiella pneumoniae* blood and respiratory isolates to meropenem and tigecycline; and of *Pseudomonas aeruginosa* respiratory isolates to imipenem, meropenem and levofloxacin. Additionally, we found significant differences in the slope of non-susceptibility trends of *Staphylococcus aureus* isolates to oxacillin and of *Enterococcus faecium* isolates to glycopeptides. Assessing in this early stage, through surveillance of routine laboratory data, the way a new global threat like COVID-19 could affect an already ongoing pandemic like AMR provides useful information for prompt action.

## 1. Introduction

Antimicrobial resistance (AMR) is globally rising and is considered an ongoing pandemic; infections caused by multidrug-resistant (MDR) bacteria contribute to an increasing number of deaths each year, with an estimated 700,000 deaths globally [1]. Moreover, a European Centre for Disease Control and Prevention (ECDC) study on the health burden of antimicrobial resistance [2] estimated that about 33,000 people die each year in the EU/EEA as a direct consequence of an infection due to MDR bacteria. The study also highlights the fact that 75% of the burden of disease is due to healthcare-associated infections (HAIs). In December 2019, an infectious disease, COVID-19, caused by the severe acute respiratory syndrome coronavirus 2 (SARS-CoV-2) was first identified in Wuhan, China, and is currently circulating throughout the world [3]. According to the World Health Organization (WHO), by 2 July 2021, there have globally been 182,319,261 COVID-19 cases, including 3,954,324 deaths [4]. In Greece, by the 5th of July 2021, 426,963 COVID-19 cases had been reported and 12,743 deaths [5]. Healthcare systems around the world are under enormous pressure, and the preparedness of different countries to tackle COVID-19 varies. In order to combat COVID-19, several changes in practices that may have impacts on AMR have taken place, absorbing huge amounts of resources from public health and healthcare systems [6]. The current pandemic forced many countries to slow down or discontinue temporarily or even postpone their national plans and other initiatives to fight AMR, re-allocating both human and budget resources to cover COVID-19 public health emergencies and duties.

In Greece, several changes in the everyday hospital routine have been recorded. Many departments, or even entire hospitals, have been transformed in COVID-19 cohort units and ICUs, while substantial changes have been recorded in the severity of cases (COVID-19 and non-COVID-19 ones) admitted in the hospitals. In Greece, as elsewhere during the pandemic, fewer planned admissions of mild cases have been recorded along with increased emergency admissions of severe COVID-19 and/or non-COVID-19 cases, especially during the second and third waves of the pandemic. On the other hand, re-allocation of human resources from non-COVID-19 hospitals or units or the private health sector to COVID-19 hospitals or units has been provisioned in order to support the overwhelmed staff. Moreover, restrictions on visitors have been implemented in all hospitals. Of note, the official high-level policies and guidelines on antibiotic stewardship in the hospital sector have not been changed during the pandemic, following the international guidelines from ECDC and WHO.

WHO issued guidance to discourage antibiotic therapy or prophylaxis for patients with mild COVID-19 symptoms or patients with suspected or confirmed moderate COVID-19 illness, unless there is clinical indication for a bacterial infection [7]. However, growing literature shows excess use of antibiotics in the treatment of COVID-19 [8,9]. As Monnet et al. state [6], the impact of the COVID-19 pandemic on AMR will only become clear when data gradually become available through national and international surveillance systems.

Surveillance is an important cornerstone to combat AMR, as has been stated in the recent World Health Organization AMR action plans [10], and routine antimicrobial susceptibility data are considered a major resource for continuous, passive AMR surveillance. Greece has been among the first countries with an electronic network based on routine susceptibility results since 1995. The Greek AMR surveillance system (WHONET-Greece) allows for continuous monitoring at the national level of antimicrobial resistance in Greek hospitals. It uses the WHONET software [11] to facilitate the collection, harmonization and analysis of routine susceptibility data from either the Laboratory Information System (LIS) or the automated antimicrobial susceptibility testing systems of the participating hospitals. The data are publicly available (www.mednet.gr/whonet, accessed on 18 August 2021) and have been continuously submitted both to the European Antimicrobial Resistance Surveillance Network (EARS-net) (https://ecdc.europa.eu/en/about-us/partnerships-and-networks/disease-andlaboratory-networks/ears-net, accessed on 18 August 2021) and to the Global Antimicrobial Resistance and Use Surveillance System (GLASS) (https://www.who.int/initiatives/glass, accessed on 18 August 2021) as the annual Greek AMR data.

In the present study, we aimed to assess the possible impact of the changes in hospitals’ daily practice due to COVID-19 pandemic on antimicrobial resistance (AMR) as captured by the WHONET-Greece AMR surveillance network.

## 2. Materials and Methods

The study covered the three-year period from January 2018 to March 2021 (2021Q1). Nine (9) out of the fourteen (14) tertiary Greek hospitals, appointed as reference hospitals for COVID-19 from the beginning of the pandemic, contributed to the study. All nine hospitals have been consistently reporting data to the WHONET-Greece AMR surveillance network, with five of them being university hospitals. The participating hospitals were distributed across the country, representing all 7 Regional Health Directorates of Greece.

The antimicrobial susceptibility testing was performed in the hospitals’ clinical laboratories by automated antimicrobial susceptibility testing systems and/or the appropriate AST method for specific antibiotics, such as Broth Microdilution Method for the MIC determination of colistin for Gram-negative bacilli. All participating hospital laboratories performed internal quality control, and they participated in the annual external quality assessment offered by ECDC–EARS-net.

During the 3-year period, routine susceptibility data of 17,837 Gram-negative and Gram-positive bacterial isolates from blood and respiratory specimens of hospitalized patients in the participating tertiary hospitals, representing the most clinically important species, were gathered and studied (Table 1). From each patient, only the first isolate of a given species recovered during the investigated time interval was included, regardless of susceptibility profile, body source or specimen type.

The classification of the isolates as susceptible, intermediate or resistant was based on either the Clinical & Laboratory Standards Institute (CLSI) [12] or the European Committee on Antimicrobial Susceptibility Testing (EUCAST) guidelines [13], depending on the AST interpretation system each hospital was using during the study period. The isolates with intermediate susceptibility were grouped with the resistant ones, forming the non-susceptible group. The data from Intensive Care Units (ICUs) were analyzed separately from medical and surgical wards, which formed the Wards group.

For every assessment period (defined as a quarter), we determined the non-susceptibility rate (number of non-susceptible isolates divided by the number of isolates tested) (Table S1). The analysis was stratified by organism, ward type (wards, ICUs), specimen type (blood, respiratory) and antibiotic.

Once the non-susceptibility rate was calculated for every quarter from January 2018 to March 2021, the data were split into the COVID-19 year (April 2020–March 2021) and pre-COVID-19 years (January 2018–March 2020). Data were then analyzed using an interrupted time-series design in order to assess the absolute and relative changes in the outcome of interest: a change in level and a change in trend. Change in level corresponds to the difference between the observed level of non-susceptibility during the first timepoint of the COVID-19 period (2nd semester 2020) versus the expected level for that timepoint, which is the level of non-susceptibility predicted by our model based on the available pre-COVID-19 period data. A change in trend corresponds to the difference in the trend (change of non-susceptibility rate over time) between “pre-interruption” and “post-interruption” periods [14].

All analyses were undertaken in Stata for Windows (v. 14.2). A *p*-value of <0.05 was considered significant.

## 3. Results

**Figure 1:**
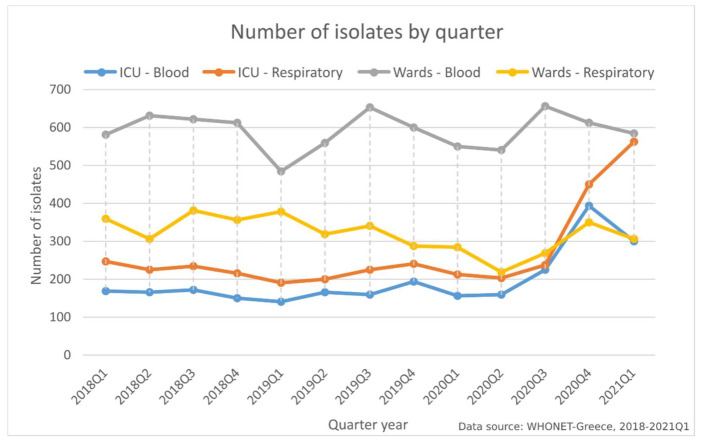
Number of bacterial isolates from blood and respiratory specimens, per quarter, from patients hospitalized in wards and ICUs of the participating hospitals, Greece, 2018–2021Q1; *x*-axis labels—year and quarter of isolation, e.g., “2018Q1” first quarter of 2018.

**Figure 2:**
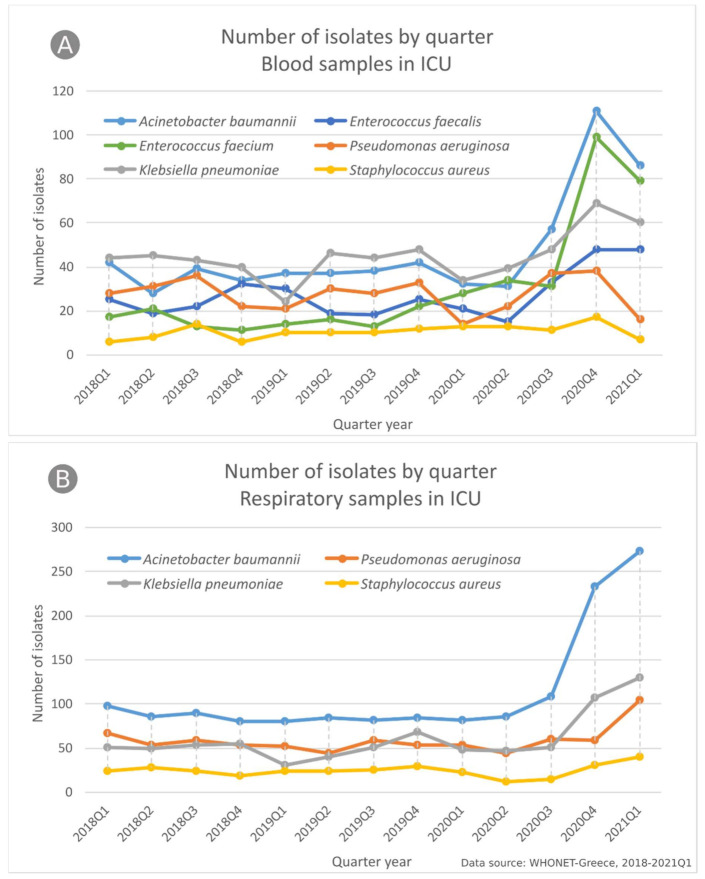
Number of bacterial isolates from (**A**) blood and (**B**) respiratory specimens, per quarter, and species, from patients hospitalized in the ICU of the participating hospitals, Greece, 2018–2021Q1; *Enterococcus faecalis and Enterococcus faecium* in (**B**) and *Escherichia coli* in (**A**,**B**) are omitted, due to very low number of isolations; *x*-axis labels—year and quarter of isolation, e.g., “2018Q1” first quarter of 2018.

Investigating the observed increase in the number of bloodstream and respiratory isolates from ICU patients in the last six months of the study period (October 2020–March 2021) (Figure 1), we found that this increase was mainly due to *A. baumannii* isolates in both blood (1.24× increase) (Figure 2A) and respiratory (1.6× increase) specimens (Figure 2B) and *E. faecium* blood isolates (1.74× increase) (Figure 2A) compared to the previous six months (April 2020–September 2020).

### 3.1. Acinetobacter baumannii

**Figure 3:**
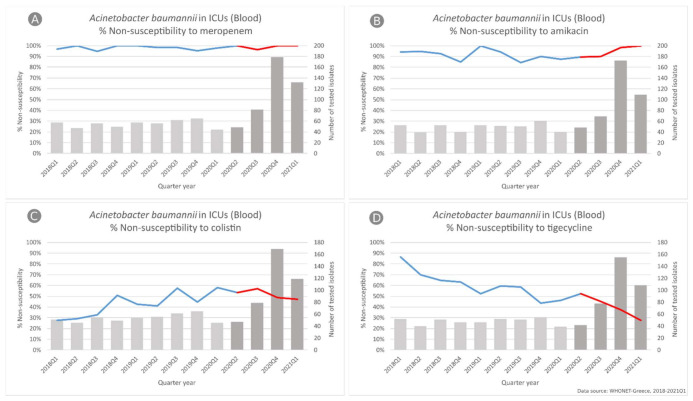
Rates (%) of non-susceptible *Acinetobacter baumannii* isolates from blood specimens to (**A**) meropenem, (**B**) amikacin, (**C**) colistin and (**D**) tigecycline, per quarter, from patients hospitalized in ICUs of the participating hospitals, Greece, 2018–2021Q1.

In *A. baumannii* blood isolates from hospitalized patients in ICU (Figure 3), we did not find any difference in the slope of carbapenem non-susceptibility trend, since it was found to consistently be very high during the whole study period, ranging for meropenem from 96.6% in the first quarter of 2018 to 100% in the first quarter of 2021.

On the other hand, we found a significant difference in the slope of the non-susceptibility trend for amikacin (*p* < 0.001), changing from a decreasing trend during the period 2018–2019 (from 94% to 87.5%, *p* = 0.007) to an increasing one during the pandemic period of the study (from 89.6% to 100%, *p* < 0.001), as well as for colistin (*p* < 0.001), changing from an increasing trend during the pre-pandemic period (from 27.5% to 57.8%, *p* < 0.001) to a decreasing one during the pandemic period (from 53% to 47%, *p* = 0.01). Tigecycline non-susceptibility rates followed a decreasing trend throughout the study period (from 86.5% to 27.5%, *p* < 0.001).

**Figure 4:**
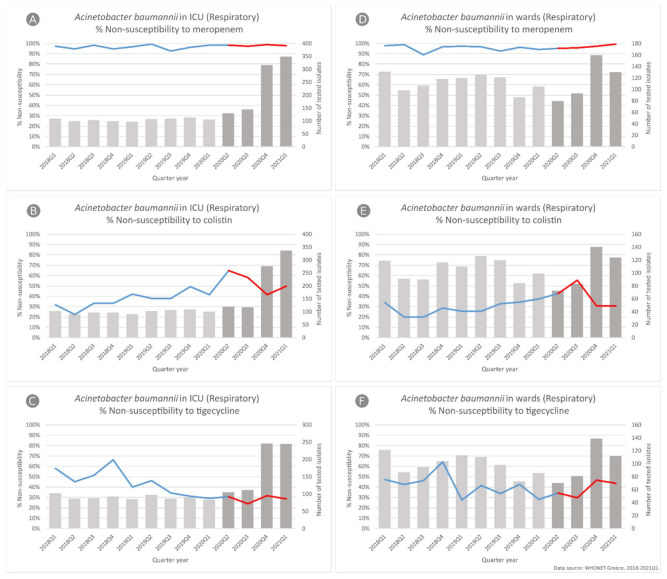
Rates (%) of non-susceptible *Acinetobacter baumannii* isolates from respiratory specimens, to (**A**) meropenem, (**B**) colistin and (**C**) tigecycline, from patients hospitalized in ICUs, and to (**D**) meropenem, (**E**) colistin and (**F**) tigecycline, from patients hospitalized in wards, of the participating hospitals, per quarter, Greece, 2018–2021Q1.

In *A. baumannii* respiratory isolates from ICU patients (Figure 4A–C), we found a significant difference in the slope of non-susceptibility for tigecycline (*p* = 0.009), changing from a decreasing trend during the pre-COVID-19 period (from 57.8% to 29%, *p* = 0.002) to a stable rate (from 30.5% to 28.7%) during the COVID-19 one. For colistin, the pattern was the same as in the blood isolates with a significant difference in the slope of non-susceptibility (*p* = 0.002), as the increasing trend during the pre-pandemic period (from 31.7% to 41.6%, *p* = 0.001) was followed by a decreasing trend (from 65% to 50%, *p* = 0.01).

In *A. baumannii* respiratory isolates from hospitalized patients in the wards (Figure 4D–F), we did not find any difference in the slope of carbapenem non-susceptibility trend, since it was found to consistently be very high during the whole study period, ranging for meropenem from 97.7% in the first quarter of 2018 to 99.2% in the first quarter of 2021.

On the other hand, we found significant difference in the slope of the non-susceptibility trend for colistin (*p* < 0.001), changing from an increasing trend during the pre-pandemic period (from 33.6% to 37.4%, *p* = 0.04) to a decreasing trend during the pandemic period (from 42.5% to 30.6%, *p* < 0.001), as well as for tigecycline (*p* = 0.001) since the non-susceptibility decreasing trend during the pre-COVID-19 period (from 47.1% to 27.9%, *p* = 0.019) was followed by an increasing trend (from 34.3% to 43.8%, *p* < 0.001) during the COVID-19 one.

### 3.2. Klebsiella pneumoniae

**Figure 5:**
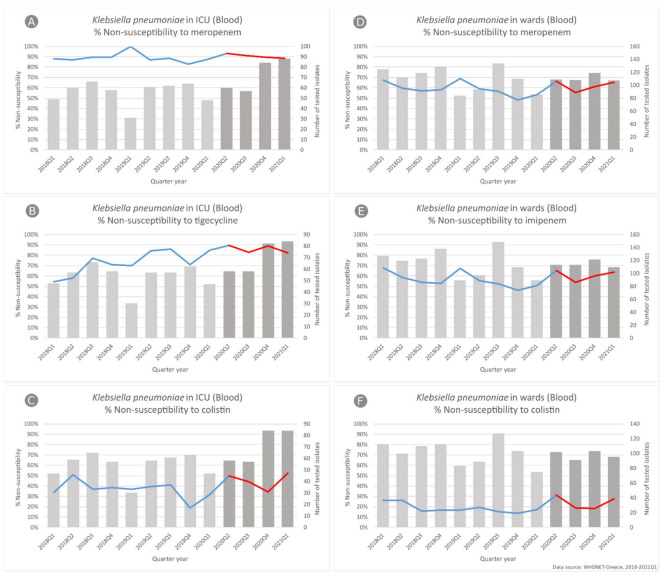
Rates (%) of non-susceptible *Klebsiella pneumoniae* isolates from blood specimens to (**A**) meropenem, (**B**) tigecycline and (**C**) colistin, from patients hospitalized in ICUs, and to (**D**) meropenem, (**E**) imipenem and (**F**) colistin, from patients hospitalized in wards of the participating hospitals, per quarter, Greece, 2018–2021Q1.

In *K. pneumoniae* blood isolates from ICU patients (Figure 5), we did not find any difference in the slope of carbapenem non-susceptibility trend, since it was found to consistently be high during the whole study period, ranging for meropenem from 87.8% in the first quarter of 2018 to 88.6% in the first quarter of 2021. On the other hand, we observed a significant change in the slope of non-susceptibility to tigecycline (*p* < 0.001), changing from an increasing trend (from 54% to 85%, *p* = 0.005) during the period 2018–2019 to a decreasing one (from 89.7% to 82%, *p* = 0.014).

For colistin, our main finding was the significant difference in the level of non-susceptibility (observed vs. expected according to our predictive model) in the second quarter of 2020 with an increased value by 16.9% (*p* = 0.031). For *K. pneumoniae* blood isolates from wards, we also found a significant difference in the level of non-susceptibility for both colistin (13.2% increase *p* = 0.008) and imipenem (13.3% increase, *p* = 0.016). In all cases, this statistically significant higher level of non-susceptibility observed in 2020Q2 seems to be maintained throughout the COVID-19 period.

**Figure 6:**
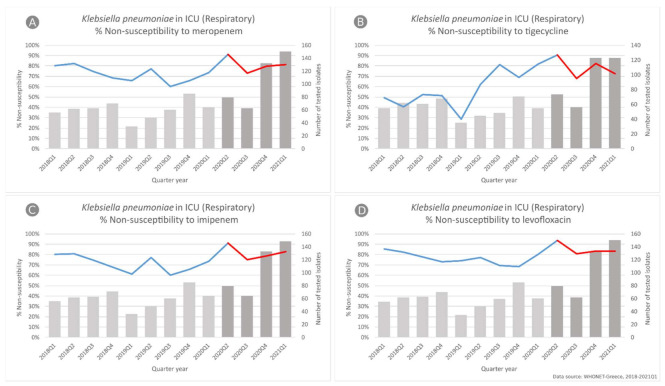
Rates (%) of non-susceptible *Klebsiella pneumoniae* isolates from respiratory specimens to (**A**) meropenem, (**B**) tigecycline, (**C**) imipenem and (**D**) levofloxacin, per quarter, from patients hospitalized in ICUs of the participating hospitals, Greece, 2018–2021Q1.

In *K. pneumoniae* respiratory isolates from ICU patients (Figure 6), we found a significant change in the slope of non-susceptibility to tigecycline (*p* = 0.003), changing from an increasing trend (49.1% to 81.8%, *p* = 0.002) to a decreasing one (from 90.5% to 72.4%, *p* = 0.026). For meropenem, imipenem and levofloxacin, our main finding was the significant difference in the level of non-susceptibility (observed vs. expected according to our predictive model) in the second quarter of 2020 with a value increased by 20.8% (*p* = 0.001), 21.6% (*p* = 0.001) and 19.5% (*p* = 0.001), respectively. In all cases, this statistically significant higher level of non-susceptibility observed in 2020Q2 seems to be maintained throughout the COVID-19 period.

### 3.3. Pseudomonas aeruginosa

In *P. aeruginosa* blood isolates from both ICU and wards as well as respiratory isolates from patients hospitalized in wards, we did not find any statistically significant changes in the slope of the non-susceptibility trends between the two periods. The median non-susceptibility for the entire 3-year period for blood isolates from ICU and wards was found for meropenem 50% and 35%, for amikacin 37.5% and 30% and for levofloxacin 53% and 48%, respectively. For *P. aeruginosa* respiratory isolates from patients hospitalized in the wards, the median non-susceptibility for the entire 3-year period was 47.1% for meropenem and 52.8% for levofloxacin.

**Figure 7:**
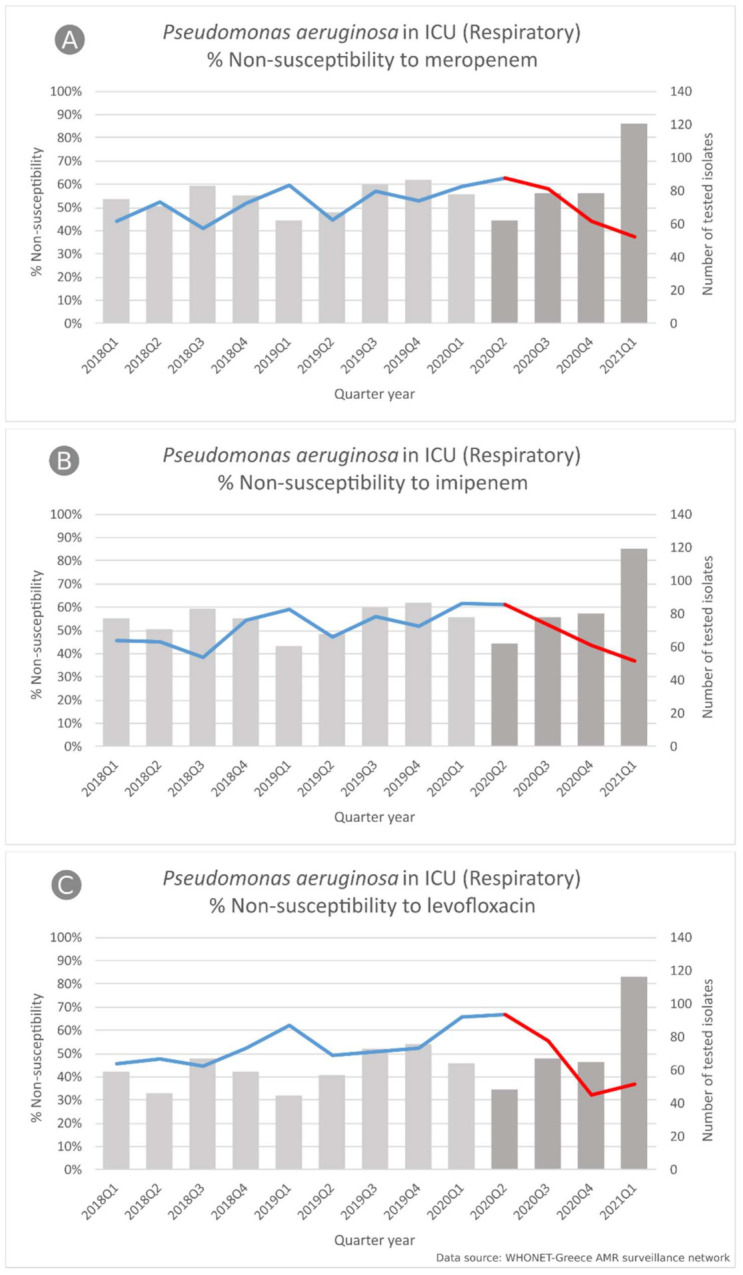
Rates (%) of non-susceptible *Pseudomonas aeruginosa* isolates from respiratory specimens to (**A**) meropenem, (**B**) imipenem and (**C**) levofloxacin, per quarter, from patients hospitalized in ICUs of the participating hospitals, Greece, 2018–2021Q1.

However, in *P. aeruginosa* respiratory isolates from ICU patients (Figure 7), we found significant changes in the slope of the non-susceptibility trend for carbapenems and levofloxacin between the two periods (all, *p* < 0.001) since the increasing trends in non-susceptibility during the pre-pandemic period for imipenem (from 45.5% to 61.5%, *p* = 0.002), meropenem (from 44% to 59%, *p* = 0.001) and levofloxacin (from 45.8% to 65.6%, *p* < 0.001) were followed by decreasing trends during the pandemic period (from 61.3% to 37%, *p* < 0.001, from 62.9% to 37.2%, *p* < 0.001, and from 66.7% to 37.1%, *p* < 0.001, respectively).

### 3.4. Enterococcus faecium

**Figure 8:**
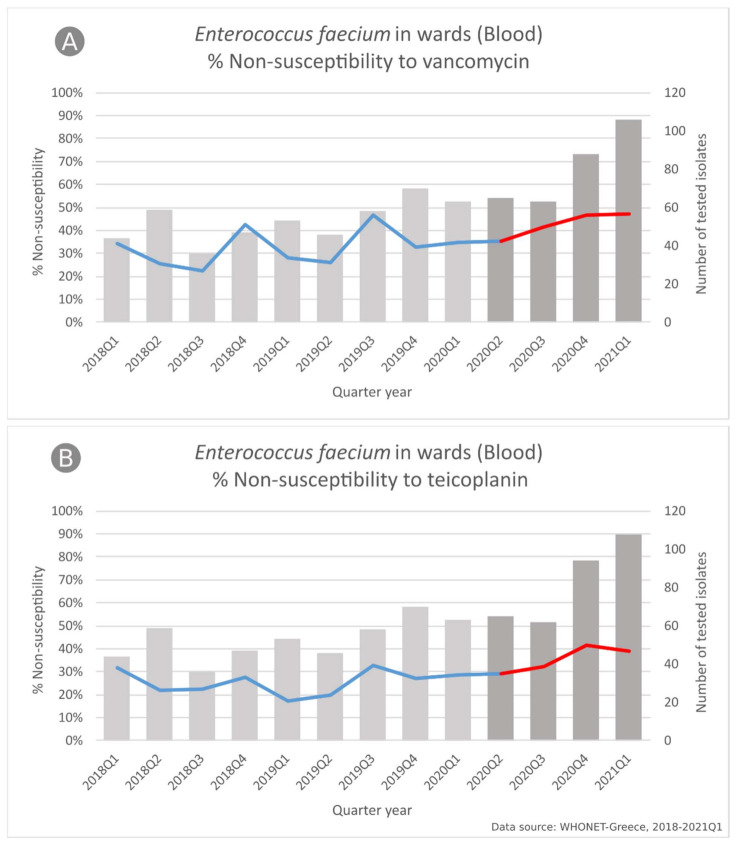
Rates (%) of non-susceptible *Enterococcus faecium* isolates from blood specimens to (**A**) vancomycin, and (**B**) teicoplanin, per quarter, from patients hospitalized in wards of the participating hospitals, Greece, 2018–2021Q1.

In *Enterococcus faecium* blood isolates from patients hospitalized in the wards (Figure 8), we found significant changes in the slope of the non-susceptibility trend for glycopeptides (for vancomycin *p* = 0.009 and for teicoplanin *p* = 0.001), since the stable non-susceptibility trend during the pre-pandemic period was followed by an increasing trend for both vancomycin and teicoplanin (from 35.4% to 47.2% and from 29.2% to 38.9%, respectively, both *p* < 0.001).

### 3.5. Staphylococcus aureus

**Figure 9:**
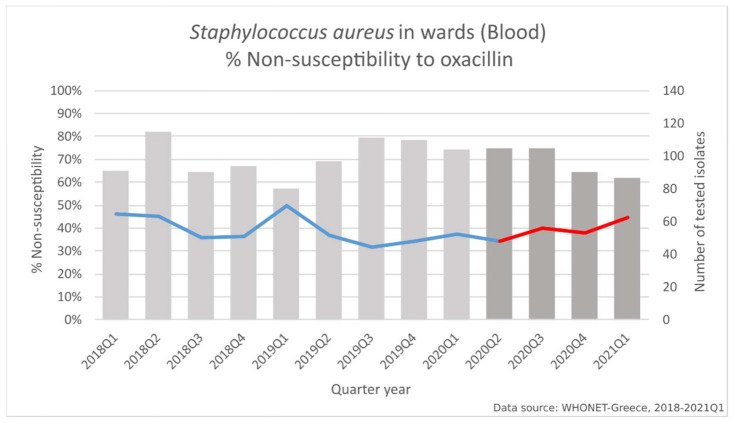
Rates (%) of non-susceptible *Staphylococcus aureus* isolates from blood specimens to oxacillin, per quarter, from patients hospitalized in wards of the participating hospitals, Greece, 2018–2021Q1.

In *Staphylococcus aureus* blood isolates from patients hospitalized in the wards (Figure 9), we found significant changes in the slope of non-susceptibility to oxacillin (*p* < 0.001), between the two study periods. Of note, the decreasing trend for oxacillin non-susceptibility during the pre-pandemic period (from 46% to 37.5%, *p* = 0.015) was followed by an increasing one (from 34.3% to 44.8%, *p* < 0.001)

## 4. Discussion

It is well established that before the onset of the COVID-19 pandemic, antimicrobial resistance (AMR) was one of the most important priorities of public health authorities worldwide [15,16]. AMR, as a demanding challenge, must now be evaluated in a new light within an altered healthcare environment. However, for more than 18 months during the COVID-19 pandemic, its effect on AMR remains vague. As Monnet et al. stated [6], specific studies will need to be performed to assess the effect of changes in antibiotic prescribing and infection control practices due to the COVID-19 pandemic on AMR.

During the pandemic period and due to the excessive pressure on the hospitals, clinicians were forced to apply empirical treatments to treat bacterial infections from common pathogens such as *E. coli*, *K. pneumoniae*, *A. baumannii*, *P. aeruginosa, Enterobacter complex*, methicillin-resistant *S. aureus*, *P. mirabilis* and *E. faecium* without evaluating the cost on AMR [15].

As for the antibiotics used in the past 18 months, it seems that carbapenems represent the majority of the applied antibiotics in combination with antibiotics widely used against bacterial infections such as other β-lactams and aminoglycosides [17,18,19,20,21,22,23,24,25]. Azithromycin also is mentioned as the macrolide most used in combination with β-lactams [17,22,23,24,26]. Other antibiotics recorded in combination with the aforementioned, are broad-spectrum tetracyclines (doxycycline, tigecycline, minocycline) [18,27,28,29,30] and antibiotics that act against respiratory tract infections (moxifloxacin, levofloxacin, clindamycin) [18,29,31,32,33], depending on the identified bacterial infection.

A thorough search into the literature (January 2020–June 2021) revealed many studies trying to address the AMR issue in the pandemic era. The main issues refer to patients with COVID-19 who may receive antimicrobial therapy (a) without a microbiological confirmation of the bacterial co-infection [18,31,32,33,34,35,36] and (b) often in the absence of a microbiological confirmation of the diagnosis [37,38,39,40,41,42,43,44,45,46,47]. Lai et al. [24] recorded the consumption of antibiotics in January–June 2019 vs. January–June 2020 in The National Taiwan University Hospital. An increase in all tested antibiotics was observed, while the resistance rates of the selected antibiotics remained constant between the two time periods with some minor exceptions. On the other hand, Tizkam et al. [48] tested 1324 samples before and after the COVID-19 pandemic; cultures revealed that the main isolated bacteria were *E. coli, K. pneumoniae* and *P. aeruginosa*. An increase in the AMR was observed in all tested antibiotics after COVID-19, mainly in meropenem and gentamicin. The latter data, although they give significant information regarding the AMR levels before and after the pandemic period, still refer to a specific hospital that is not representative of the country’s situation. Few other studies have tried to answer the major question of how the pandemic affected AMR [25,34,49], mainly in a specific hospital level or measuring the antibiotic consumption before and during the pandemic. The comparison of AMR levels before and after the changes to hospital daily practices due to COVID-19 pandemic is not discussed in none of these studies.

To our knowledge, this is the first study of the possible impact of COVID-19 to AMR as it has been captured by a national surveillance system based on routine laboratory data. Of note, our surveillance system monitors laboratory routine susceptibility data and collects data from a variety of clinical specimens, enabling us to focus not only on bloodstream isolates but also on respiratory ones and thus capture a larger part of the AMR picture before and during COVID-19 pandemic. Furthermore, the inclusion of respiratory isolates could give an insight into the susceptibility pattern of isolates colonizing the respiratory tract or being a putative cause of co-infections or secondary infections in patients with COVID-19 to inform for optimal empirical antimicrobial treatment.

Additionally, interrupted time-series analysis is used for the first time to assess the potential differences in the level of resistance in the first quarter following the observed changes in our hospitals due to COVID-19 and the AMR change rate, before and after that time point.

Regarding to *A. baumannii* blood and respiratory isolates, an increased isolation frequency along with very high levels of carbapenem non-susceptibility throughout the study period were observed. Moreover, changes in the slope of non-susceptibility trends to other antibiotics were found along with changes from decreasing to increasing trends for amikacin and tigecycline or from an increasing trend to a decreasing one for colistin, remaining, however, at a high level (47%). *Acinetobacter baumannii* is an opportunistic pathogen with the ability to survive in hospital environments for a long time and gain many virulence factors, emerging as an important nosocomial pathogen. Several factors could have contributed to the increased isolation frequency of this pathogen during the pandemic: the increased clinical severity of hospitalized cases, the increased duration of hospitalization and the increased use of antibiotics and mainly carbapenems, mostly in the ICU setting. Already during the first wave of the pandemic, an increased incidence of ICU-acquired BSIs, mostly due to *A*. *baumannii* and *K*. *pneumoniae* followed by *Enterococcus* spp, among COVID-19 patients was reported in a COVID-19 reference hospital in Greece. *A.* *baumannii* isolates were extensively drug-resistant and pan-drug-resistant. Of note, the presence of BSIs was associated with considerable prolongation of mechanical ventilation and length of ICU stay [50].

Similar findings of multi-drug-resistant *A. baumannii* isolates have been recently mentioned elsewhere in COVID-19 patients in both blood and respiratory isolates, mainly from ICUs in high percentages [51,52]. High rates of resistance were observed in almost all widely used antibiotics in *A. baumannii* infections, such as carbapenems/meropenem [27,53,54], other β-lactams [19,55], aminoglycosides/amikacin [19,27,30,54] and colistin [52,53]. Tigecycline was also reported to be used in COVID-19 patients with *A. baumannii* co-infections; susceptible isolates reported in Li et al., 2020 [27] and Kyriakidis et al. [52]; and resistant isolates in Vijay et al. [56] and Chen et al. [30].

As for *K. pneumoniae* blood isolates from ICU patients, we found a significant difference in the slope of the non-susceptibility trend for meropenem, changing from the stable trend during the 2018–2019 period to a decreasing trend during the pandemic period (from 93% to 88.6%), remaining, however, at a high level of resistance. It is well documented that Greece has been facing high rates of carbapenem resistance among hospital *K. pneumoniae* isolates since 2002 due to mainly carbapenemase-producing strains, imposing therapeutic challenges at a clinical level [57]. Co-infection with carbapenemase-producing *K. pneumoniae* in COVID-19 patients has been reported recently from Greece [50] and other countries. Invasive infections due to multidrug resistant KPC and/or OXA-48 carbapenemase-producing *K. pneumoniae* have been reported by Dumitru et al. [58] in nine patients hospitalized in an intensive care unit (ICU) with severe COVID-19. Another review study by Medrzycka-Dabrowska et al. [59] reported COVID-19 patients with carbapenem-resistant *K. pneumoniae* isolates from six countries—Italy, China, Egypt, United States, Spain and Peru—at a prevalence ranging from 0.35% to 53%.

For colistin, which has been increasingly used in Greece since 2010 for the treatment of carbapenem-resistant *K. pneumoniae*, our main finding was the significant increase in the level of non-susceptibility, a finding similar for *K. pneumoniae* blood isolates from wards. Since colistin is one of the few treatment options for carbapenem-resistant *K. pneumoniae* infections, colistin resistance represents a challenge due to the limited range of potentially available effective antimicrobials.

In *P. aeruginosa* respiratory isolates, significant changes in the slope of the non-susceptibility trend for carbapenems, amikacin and levofloxacin between the two periods, with decreasing trends during the pandemic period, were highlighted. However, in blood isolates, we found consistently high non-susceptibility rates for the aforementioned antibiotics during both pre-pandemic and pandemic periods. *P. aeruginosa* has been identified as a common coinfecting pathogen in COVID-19 patients causing exacerbation of illness [29,56,60,61,62]. *P. aeruginosa* has shown high resistance to many antibiotics used during the COVID-period, such as carbapenems [48,63,64] and amikacin [48,65,66]. As for levofloxacin, high *P. aeruginosa* resistance levels and increased use during the COVID-19 period have been reported in the literature—[48,66] and [67,68], respectively.

As for *E. faecium,* we observed increased isolation numbers from bloodstream infections in ICU patients during the pandemic period, a finding in line with the literature [23,69,70,71,72]. Moreover, in blood isolates from patients hospitalized in wards, we found significant changes in the slope of the non-susceptibility trend for vancomycin and teicoplanin, since the stable non-susceptibility trend during the pre-pandemic period was followed by an increasing trend for both vancomycin and teicoplanin during the pandemic period. Regarding recent data on vancomycin-resistant enterococci (VRE) from Greece, a point prevalence study of VRE/Carbapenem-resistant Gram-negative (CRGN) rectal carriage of inpatients was conducted in March 2018 in one of the participating hospitals, finding 13.0% of inpatients to be positive for VRE carriage, 8.2% for CRGN and 2.1% for both VRE and CRGN. All VRE isolates were identified as VanA-phenotype *E. faecium* (high level resistance to vancomycin and teicoplanin) [73]. These findings, along with our findings on both increased *E. faecium* isolation and non-susceptibility to glycopeptides during the COVID-19 deserve close monitoring and appropriate interventions to limit their spread.

In *Staphylococcus aureus* blood isolates from patients hospitalized in the wards, significant changes in the slope of non-susceptibility to oxacillin between the two study periods was observed, which is consistent with the literature [27,65,74]. Of note, the decreasing trend for oxacillin non-susceptibility during the pre-pandemic period was followed by an increasing one.

One possible limitation of our study is the probable confounding due to the inclusion of March 2020 in the 2020Q1 quarter as part of the pre-COVID period, since during March 2020 we had our first hospitalized cases in Greece. However, we considered that the burden in our system due to the COVID-19 hospitalizations was still negligible in that time period, with the quarterly tracking showing little change from baseline in the 2020 Q1 timepoint. Another possible limitation of our analysis is the use of quarterly averages that may has imparted some degree of bias. However, the inclusion of nine hospitals and the stratification of the analysis by organism, ward type and specimen type resulted in a volume of isolates and AST data that could not be analyzed in shorter intervals.

## 5. Conclusions

Despite the aforementioned possible limitations, our study provides valuable, preliminary results on the possible impact of COVID-19 on antimicrobial resistance (AMR). Greece has been facing an endemic situation of multidrug-resistant pathogens in the hospital sector, mainly due to carbapenem-resistant Gram-negative bacilli, since the late 2000s, while, in general, the antimicrobial resistance rates are among the highest in Europe. Taking into account this difficult situation, it was of utmost importance to try to understand the interaction between the current COVID-19 pandemic with the enormous pressure that is put on our healthcare system and AMR. Our preliminary findings are indicative of an increased isolation frequency of multidrug-resistant *A. baumannii* and *E. faecium* BSIs during the COVID-19 period, constantly high-level of carbapenem resistance in *A. baumannii* and *K. pneumoniae* isolates and increased colistin nonsusceptibility in *K. pneumoniae* bloodstream and respiratory isolates. On the other hand, decreasing nonsusceptibility trends were observed in respiratory *P. aeruginosa* isolates for the most clinically relevant antibiotics. Finally, during the COVID-19 period, increasing non-susceptibility trends were found in *E. faecium* and *S. aureus* bloodstream isolates for glycopeptides and oxacillin, respectively.

In order to assess the possible impact of the changes in hospitals’ daily practice due to COVID-19 pandemic on AMR, the timely availability of data from the applied surveillance systems is of great importance. In this context, our study provides preliminary results through WHONET-Greece electronic surveillance system based on routine data. With the COVID-19 pandemic evolving, this data could serve as the basis for further studies required to better understand the impact and support decision-making for prompt interventions for future challenges.

## Figures and Tables

**Table 1:** Number of bacterial isolates from blood and respiratory samples per year and species from patients hospitalized in wards and ICUs of the participating hospitals, Greece, 2018–2021Q1.

Microorganism	Quarter of Isolation													Total	
	2018				2019				2020				2021		
	Q1	Q2	Q3	Q4	Q1	Q2	Q3	Q4	Q1	Q2	Q3	Q4	Q1	n	%
**Number of bacteria isolated in ICUs**															
*Acinetobacter baumannii*	140	113	128	114	117	121	119	126	114	116	165	344	359	2078	34.6
*Klebsiella pneumoniae*	95	95	97	95	55	86	95	116	82	86	99	176	190	1367	22.8
*Pseudomonas aeruginosa*	95	84	95	76	73	74	87	86	67	66	97	97	121	1118	18.6
*Escherichia coli*	13	23	13	14	10	16	14	15	20	11	14	22	11	196	3.3
*Enterococcus faecalis*	27	19	22	32	30	19	21	26	22	18	33	52	51	372	6.2
*Enterococcus faecium*	17	21	13	11	14	17	13	24	29	40	31	104	84	418	7.0
*Staphylococcus aureus*	30	36	38	24	34	34	35	41	36	25	25	48	47	453	7.5
***Total in ICUs***	**417**	**391**	**406**	**366**	**333**	**367**	**384**	**434**	**370**	**362**	**464**	**843**	**863**	**6002**	**100.0**
**Number of bacteria isolated in wards**															
*Acinetobacter baumannii*	184	135	175	149	160	178	182	124	152	134	180	222	194	2169	18.3
*Klebsiella pneumoniae*	185	167	195	217	172	154	201	165	135	141	164	196	155	2247	19.0
*Pseudomonas aeruginosa*	163	183	213	192	163	155	189	165	136	118	174	143	138	2132	18.0
*Escherichia coli*	162	180	184	170	132	161	164	173	151	131	149	140	112	2009	17.0
*Enterococcus faecalis*	67	57	69	59	53	51	52	57	56	50	67	62	60	760	6.4
*Enterococcus faecium*	46	59	38	50	54	46	58	69	61	62	63	84	102	792	6.7
*Staphylococcus aureus*	132	157	126	132	130	132	147	135	143	123	128	114	127	1726	14.6
***Total in wards***	**939**	**938**	**1000**	**969**	**864**	**877**	**993**	**888**	**834**	**759**	**925**	**961**	**888**	**11,835**	**100.0**

## Data Availability

Data available in a publicly accessible repository http://www.mednet.gr/whonet/ (accessed on 18 August 2021).

## Supplementary Materials

The following are available online at https://www.mdpi.com/article/10.3390/life11100996/s1, Table S1, Rates (%) of non-susceptibility by organism, ward type and specimen type, for all bug-drug combinations used for this study.
